# Relationship Among Motor Behavior, Motor Development, and Motor Performance in Children Aged 7–8 Years in China

**DOI:** 10.3389/fpubh.2022.898266

**Published:** 2022-05-31

**Authors:** Hongbing Zhang, Jiajia Cheng, Zongping Wang

**Affiliations:** ^1^MQ Research Center, Nanjing University of Science and Technology, Nanjing, China; ^2^College of Economics and Management, Nanjing University of Aeronautics and Astronautics, Nanjing, China; ^3^School of Economics and Management, Nanjing Tech University, Nanjing, China; ^4^College of Physical Education, Yunnan University, Kunming, China

**Keywords:** motor behavior, motor development, motor performance, physical activity, health, motional quotient

## Abstract

For children aged 7–8 years in China, “immobility” is a key problem hindering their physical and mental development in recent years. It is widely accepted that motor performance development in children is accompanied by physical and mental growth and development. However, few studies have clarified the relationship among motor behavior, motor development and motor performance. To bridge this knowledge gap, an empirical analysis of children aged 7–8 years in China was conducted. This study developed scales for testing motor performance, motor behavior and motor performance, respectively, and collected data of these tests on children aged 7–8 years in China. Canonical correlation analysis was used to analyze the correlations among motor performance, motor behavior and motor performance, and partial least squares regression was used to evaluate the relationship between dependent and independent variables. It was found that, for the children aged 7–8 years in China, there were significant positive correlations among the motor performance, motor behavior, and motor development. The three tests were closely related and could be applied to promote children's sports performance through improved training activities for targeting specific indicators. The study found there was no significant differences in the application of the three tests for children with different age and gender. This finding lays a foundation for further testing in older children and meets the measurement requirements of modern medicine's “bio-psycho-social model of health promotion”. Additionally, the theoretical motional quotient model of “The Bio-Behavior-Task” is constructed as a comprehensive motor performance evaluation system, aligning with students' physical and mental development standards.

## Introduction

Physical inactivity and sedentary behavior issues are increasing in modern society, carrying health risks for humans, i.e., disease, disability, and even death (1). In childhood, this poor lifestyle manifests as a lack of exercise ability, insufficient physical activity, increased risk during exercise behavior, stiff and weak movement, motor retardation, a high obesity rate, poor social adaptation, and even cardiovascular-related diseases, etc. (2, 3). For Chinese children aged 7–8 years, “immobility” is also a key problem affecting their physical and mental health development.

Only 8.9% of children and adolescents engage in 1 h of high-intensity physical exercise more than three times a week. According to the eight Reports on the Physical Fitness and Health Research of Chinese School Students from 1985 to 2019, the living standards have improved significantly, while the physical health conditions in children have not increased as expected. What's worse, the physical fitness level specific to endurance, strength, and speed have continued to decline (4). For promoting the physical health conditions, Chinese government has implemented annual physical health test for children students. However, the uniformly-used national standard for students' physical health test ignores the concepts of individual development, environmental impact, experience acquisition and sports development, rendering it impossible to formulate educational and practical programs based on the test. Thus, developing the scales for testing motor behavior, motor development, and motor performance, respectively, and clarifying their relationships is highly needed for the children's physical health test.

Along with the motor development, the structure and function of human tissues and organs change over time, which directly leads to changes in motor performance (5–7). Accordingly, the physical education in China has emphasized being “in line with the law of students' physical and mental development”, while the understanding of this developmental relationship is still in the preliminary exploration and empirical speculation stage. Additionally, with the goal of promoting the children's physical health, previous studies have focused on children's physical activity, physical fitness, motor performance, physical literacy, sports intelligence, sports behavior, movement development, fitness exercise, behavioral risk, physical fitness monitoring, and health promotion (8–17). For example, Phillip (14) and David (15) found that regular physical activity was an important factor for promoting health at any age (18, 19). Newell (5) proposed an action development model for “how individuals, environment and action tasks interact” based on social ecology. However, most of these studies ignore the effect of environment and task on evaluating physical activities and behaviors of an individual (20). Namely, few studies clarified the relationship among motor behavior, motor development, and motor performance.

To bridge the aforementioned research gaps, this study aims to explore the relationship among motor performance, motor behavior and motor development, and facilitate their cooperative development for children. Targeting at the children aged 7–8 years, we firstly developed the scales for testing the children's motor behavior, motor development, and motor performance, respectively, on basis of the requirements for health promotion education and environmental support conducive to health change. After collecting the test data, we analyzed the results of surveyed children's motor performance, motor behavior and motor development, and constructed the comparative analysis in consideration of the variance in children's age and gender. Finally, this study further discussed several specific findings in relation to correlation analysis, impact relationship analysis, and the development of the Motional Quotient scale as well as its theoretical model.

## Materials and Methods

### Sample Design and Selection

After the literature review and policy analysis on the existing physical health tests, this study conducted an expert interview to develop the scales for testing the children's motor performance, motor behavior and motor development. Twelve experts (three researchers with more than 3 years' professional experience in the field of physical health and behavior development, three researchers with more than 5 years' professional experience in the field of children psychology, three researchers with more than 3 years' professional experience in the field of behavior development, and three experts with more than 5 years' working experience in motor development) were invited to be interviewed together *via* the on-line meeting. Each interviewed expert will be asked several open questions, such as “please show your opinion about the relationship between the children's motor performance and their physical and mental growth and development,” “please explain your opinion in detail,” “please show your opinion about our primarily selected items for testing the motor performance, motor behavior, and motor development,” and “please list the concerns during the test”. Notably, during the interview, we firstly provided the list of the primarily selected test items regarding the motor performance, motor behavior, and motor development, which we have selected *via* systematic literature review and policy analysis. All the experts confirmed the relationship between the children's motor performance and their physical and mental growth and development, and they finally achieved consistence for the scales of testing motor performance, motor behavior, and motor development through discussion. That is, the initial three test scales were developed.

Based on the preliminarily determined test scales, we provided a series of supporting files to the testers and testees, including test manual, test demonstration video, and scale recording form. Notably, these supporting files were confirmed by the invited 12 experts. Prior to the test, we performed the unified training for the testers regarding the test methods. During the test, the tester first presented one-time correct demonstration for each tested item, and then the testee practiced one time. Each testee performed the formal test one time for each tested item, and the testers recorded the test results. The formal test period was March 2018 to September 2020.

For performing the pretest, 10 boys and 10 girls in each age group (aged 7 and 8 years) were randomly selected in Jiangsu, China, for conducting the pretest. According to the pre-test results and the testees' feedback, the test scales for each dimension, i.e., motor performance, motor behavior, and motor development, were revised appropriately.

For performing the formal test, a total of 400 children testees aged 7–8 years were randomly selected from 4 provinces (cities) in China (Jiangsu, Shanghai, Zhejiang, and Shandong). There were 100 boys and 100 girls in the testee group of aged 7 years and aged 8 years, respectively.

### Variables and Rating Scale

#### Motor Performance

It refers to the ability of an individual to perform a physical motor skill, which comprehensively representing the individual body shape, physical function, and physical qualities. Following the National Standard for Students' Physical Health (21), FITNESSGRAM (22), and ACSM (American College of Sports Medicine) guidelines for exercise testing and prescription (23), several test items relating to BMI, speed, strength, endurance, flexibility, sensitivity and balance were selected to represent the motor performance dimension. All these test items were summarized in Table 1.

**Table 1:** Motor performance scale.

**No**.	**Item**	**Unit**	**No**.	**Item**	**Unit**
x1	Height	cm	x5	Rope skipping (1 min)	count
x2	Weight	kg	x6	Sitting body flexion	cm
x3	Turn back run (2 * 30 m)	s	x7	Throw solid ball in place (1 kg)	m
x4	Plate support	s	x8	Reverse run (20 meters)	s

#### Motor Behavior

It's aim is to explore the environmental interaction experience of the individuals when they participated in sports, including the measures of motor behavior, motor motivation, sports performance, and sports cognition. Based on the systematic review of the IPAQ (International Physical Activity Questionnaire) (24, 25), AHKC (Active Healthy Kids Canada) (26); internal motivation questionnaire, motor behavior scales, motor motivation scales; sports situational motivation scales, exercise attitude scales, tennis performance evaluation scales, sports performance strategy scales, athlete stress scale (27), and CAPL (Canadian Assessment of Physical Literature) (12), this study developed the motor behavior scales relating to daily exercise behavior (28), motivation and the psychological experience of participating in sports, psychological skills and applied strategies in sports, and basic knowledge and understanding of sports literacy. All these test items were summarized in Table 2. Notably, the motor behavior test was conducted through question-and-answer format.

**Table 2:** Motor behavior scale.

**No**.	**Item**	**No**.	**Item**
y11	Average daily steps over 28 days	y21	Effective strategies are used in sports
y12	Number per week of medium and high intensity exercise sessions of more than 1 h	y22	Moderate activities strengthen the will and regulate sleep
y13	Number per week of interactive activities of more than 20 min	y23	Recognize that you have shortcomings in sports
y14	More than 10 min of action/skill learning per week	y24	Exercise only under the influence of partners, and do not take the individual initiative to exercise
y15	Skill at 1–2 sports to meet individual needs	y25	Ability to concentrate on activities
y16	People who feel that they are good at sports are popular	y26	Examples of health knowledge
y17	Fear of injury in sports games	y27	Understanding of physical activity
y18	Sports games are more interesting than computers (mobile phones, TV)	y28	Understanding of actions/skills
y19	You can learn movements more easily	y29	Understanding of activity time and environment
y20	Cannot improve the exercise level in the necessary time	y30	Understanding of body posture

*The item y17, y20, and y24 were reverse items with Five-Likert scale*.

#### Motor Development

It is used to test the ability of individuals to complete motor tasks. Through developmental evaluation, such as motor development tests, motor skill improvement and motor function evaluation during individual development (29), we can scientifically understand the relation of motor development to the physical and mental growth and development of Chinese children. With reference to the PMDS (Peabody Motor Development Scale) (30), and TGMD (Testing of Big Muscle Group Development) (31), the test items of motor development include posture, operation, hand-eye coordination, and reaction (32–34). In specific, the posture (Item z31), being widely-used by NASM (National Academy of Sports Medicine, USA), is a classic action for evaluating dynamic posture, with which the dynamic flexibility and muscle control are assessed. The operation (Item z32) include tapping, catching, kicking, throwing and dribbling, aiming to testing the gross movement development. The hand-eye coordination (Item z33) includes swinging, rotating, bouncing, catching, and throwing for testing the fine movement development. According to the characteristics of children's periodically development, we developed the test items in relation to imitation, specific skills static control, and dynamic scenes. Specifically, behavior (Item z34) is the performance of life experience acquisition, specific skills (z35) involve changes in physical education and learning, the squat control (z36) is the effect of fitness and training, and dynamic scenario (z37) is the intelligent level of action processing. The reaction actions (Item z38) consists of walking on a balance beam, stepping on a five-pointed star, crawling in all directions and rotating in place to test the abilities of movement, climbing and balance. The detailed action procedure and scoring criteria for each item of the motor development scales are provided in Appendix 1, and the corresponding schematic figures are provided in Appendix 2.

### Statistical Analyses

The collected data were analyzed by SPSS 28.0. The significance level of the hypothesis test using a two-sided test was 0.05. Canonical correlation analysis was used to analyze correlations, and the Spearman correlation coefficient was used to evaluate the possible correlations among the dimensions of motor performance, motor behavior, and motor development. Partial least squares regression was used to evaluate the relationships between dependent and independent variables.

## Results

### Test Results

The mean ± standard deviation for each tested item (x1-x8, y11-y30, and z31-z38) regarding the motor performance, motor behavior, and motor development of the children with different age and gender are shown in Table 3.

**Table 3:** Test results categorized by the age and gender.

**No**.	**Item**	**7 years old**		**8 years old**	
		**Boys (*n* = 100)**	**Girls (*n* = 100)**	**Boys (*n* = 100)**	**Girls (*n* = 100)**
		**Mean ±SD**	**Mean ±SD**	**Mean ±SD**	**Mean ±SD**
x1	Height	125.56 ± 4.93	123.17 ± 5.12	131.27 ± 4.76	129.46 ± 6.04
x2	Weight	25.72 ± 5.12	24.36 ± 5.85	30.68 ± 7.12	26.90 ± 5.95
x3	Turn back run (2 * 30 m)	15.79 ± 1.39	16.49 ± 1.48	14.80 ± 0.89	15.61 ± 1.12
x4	Plate support	19.12 ± 10.92	9.92 ± 5.29	27.52 ± 13.33	29.76 ± 13.81
x5	Rope skipping (1 min)	17.18 ± 16.12	9.48 ± 9.28	53.24 ± 32.15	60.38 ± 31.63
x6	Sitting body flexion	7.86 ± 3.68	10.86 ± 4.26	7.59 ± 3.45	11.50 ± 3.92
x7	Throw solid ball in place (1 kg)	2.35 ± 0.63	1.74 ± 0.55	3.15 ± 0.59	2.26 ± 0.57
x8	Reverse run (20 m)	11.90 ± 1.66	10.74 ± 1.58	9.79 ± 1.44	10.90 ± 1.82
y11	Average daily steps over 28 days	3.46 ± 1.11	3.32 ± 1.10	3.44 ± 1.30	3.72 ± 1.18
y12	Number per week of medium and high intensity exercise sessions of more than 1 h	3.46 ± 1.01	3.50 ± 0.99	3.48 ± 1.09	3.52 ± 0.99
y13	Number per week of interactive activities of more than 20 min	3.34 ± 1.02	3.26 ± 1.01	3.98 ± 0.96	3.74 ± 1.16
y14	More than 10 min of action/skill learning per week	3.04 ± 1.12	3.30 ± 1.22	3.94 ± 1.88	3.58 ± 1.09
y15	Skill at 1–2 sports to meet individual needs	4.10 ± 1.07	3.98 ± 1.12	4.20 ± 1.09	4.20 ± 0.97
y16	People who feel that they are good at sports are popular	4.42 ± 1.11	4.32 ± 1.04	3.84 ± 1.15	4.00 ± 1.14
y17	Fear of injury in sports games^®^	2.04 ± 1.47	2.48 ± 1.53	1.92 ± 1.19	2.14 ± 1.29
y18	Sports games are more interesting than computers (mobile phones, TV)	4.30 ± 1.33	4.16 ± 1.40	3.78 ± 1.37	3.82 ± 1.40
y19	You can learn movements more easily	4.06 ± 0.96	4.00 ± 1.03	3.86 ± 1.07	4.12 ± 0.94
y20	Cannot improve the exercise level in the necessary time^®^	2.76 ± 1.60	2.72 ± 1.41	2.50 ± 1.49	2.54 ± 1.37
y21	Effective strategies are used in sports	3.98 ± 1.52	3.60 ± 1.59	3.44 ± 1.53	3.36 ± 1.50
y22	Moderate activities strengthen the will and regulate sleep	4.30 ± 1.20	4.54 ± 0.99	4.14 ± 1.16	4.16 ± 1.15
y23	Recognize that you have shortcomings in sports	3.22 ± 1.71	3.36 ± 1.59	3.80 ± 1.32	3.96 ± 1.26
y24	Exercise only under the influence of partners, and do not take the individual initiative to exercise^®^	2.28 ± 1.67	2.32 ± 1.66	2.26 ± 1.55	1.92 ± 1.44
y25	Ability to concentrate on activities	4.08 ± 1.31	4.02 ± 1.22	4.04 ± 1.26	4.06 ± 1.19
y26	Examples of health knowledge	3.92 ± 1.21	4.14 ± 1.13	4.24 ± 0.85	4.20 ± 1.03
y27	Understanding of physical activity	3.38 ± 1.35	4.04 ± 1.07	3.86 ± 1.20	3.72 ± 0.95
y28	Understanding of actions/skills	3.40 ± 1.21	3.72 ± 1.07	3.74 ± 1.10	3.60 ± 1.16
y29	Understanding of activity time and environment	3.26 ± 1.21	3.42 ± 1.33	3.42 ± 1.20	3.22 ± 1.17
y30	Understanding of body posture	3.16 ± 1.50	3.10 ± 1.40	3.60 ± 1.12	3.34 ± 1.38
z31	Posture	3.30 ± 1.02	3.36 ± 1.01	3.36 ± 0.90	3.50 ± 1.04
z32	Operation	2.54 ± 0.93	2.80 ± 0.99	2.88 ± 1.02	3.18 ± 1.08
z33	Hand-eye coordination	2.06 ± 0.74	1.78 ± 1.00	2.24 ± 0.96	1.98 ± 0.87
z34	Behavior	3.44 ± 0.91	3.34 ± 1.00	3.32 ± 0.98	3.40 ± 0.88
z35	Special skills	4.28 ± 0.97	3.80 ± 1.14	4.70 ± 0.79	4.72 ± 0.67
z36	Squat control	3.28 ± 0.97	3.20 ± 0.93	3.18 ± 0.96	3.58 ± 1.05
z37	Dynamic scenario	3.06 ± 1.00	3.38 ± 1.09	2.92 ± 1.01	3.50 ± 1.07
z38	Reaction	54.32 ± 8.06	54.70 ± 8.84	45.86 ± 6.30	49.73 ± 7.14

### Canonical Correlation Analysis

**Figure 1:**
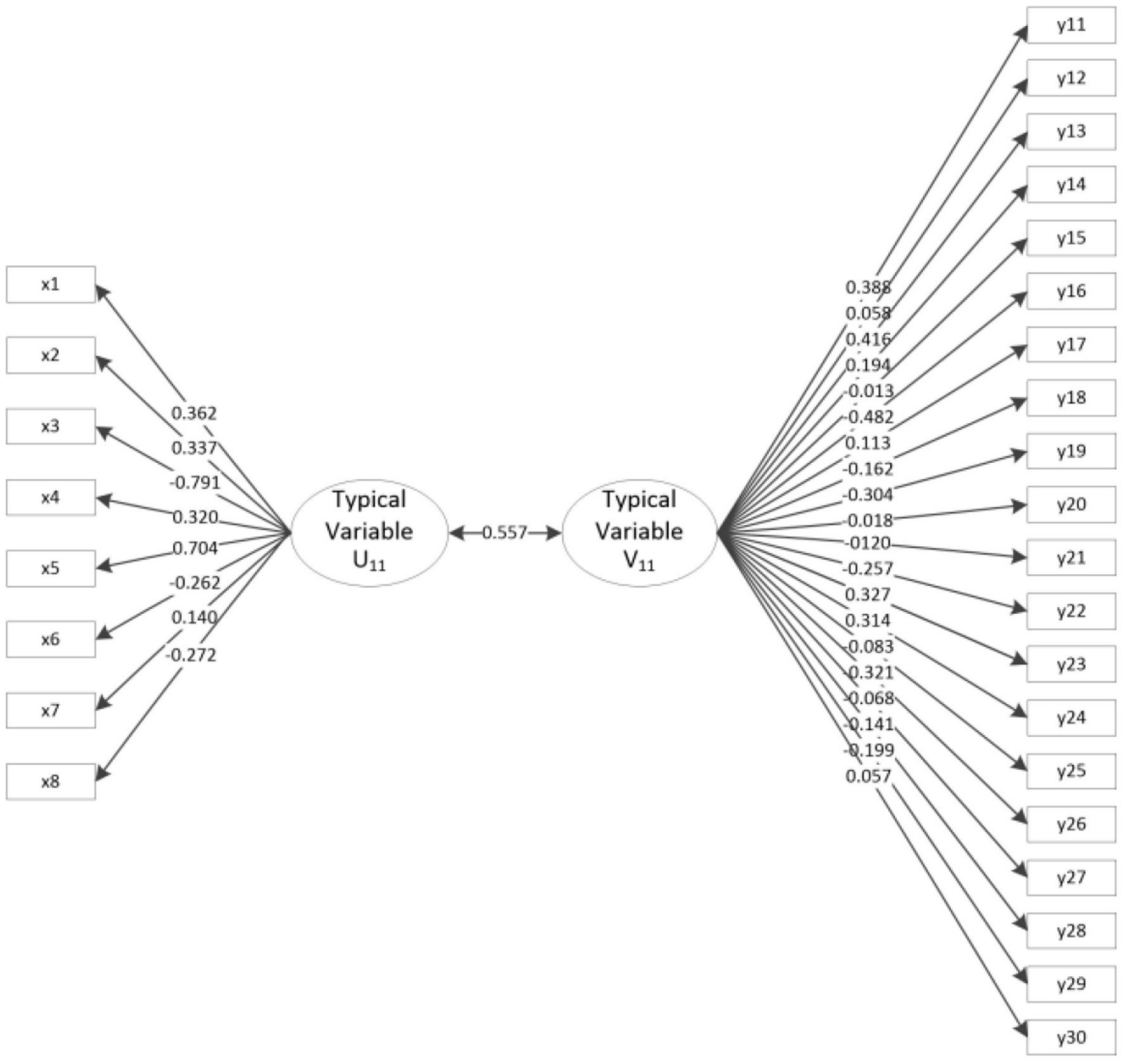
Typical load coefficient and cross-load coefficient for motor performance and motor behavior.

Table 4 and Figure 1 show the results of the canonical correlation analysis of motor performance and motor behavior tests. The results show that a total of 8 typical variables are extracted. The *F*-test indicates that the first pair of typical variables are significant at the 0.01 level with a correlation coefficient of 0.557 > 0.5, which is a very high value. There is a close positive correlation between the first pair of typical variables (U_11_ represents the first typical variable of motor performance, and V_11_ represents the first typical variable of motor behavior). Focusing on the intragroup difference analysis of the typical variables U_11_ and V_11_, the load coefficients of x3 and x5 for typical variable U_11_ and Group X are 0.791 (absolute value) and 0.704, indicating a strong relationship; that is, typical variables extract more information from x3 and x5. The load coefficients of y13 and y16 for typical variable V_11_ and Group Y are 0.416 and 0.482 (absolute value), indicating a strong relationship; that is, typical variables extract more information from y13 and y16. Therefore, the main factors of motor performance, i.e., turning back and running (x3) and rope skipping (x5), are closely related to the main factors of motor behavior, i.e., “the number per week of interactive activities of more than 20 min” (y13) and “the people who feel that they are good at sports are popular” (y16). There is a very close positive correlation between motor performance (Group X) and motor behavior (Group Y).

**Table 4:** Typical correlation coefficients and significance between motor performance and motor behavior.

**Typical variable pair**	**Canonical correlation coefficient**	**Wilks' lambda**	***df*1**	***df*2**	***F***	***p***
1	0.557	0.304	160.000	1,291.300	1.397	0.001^**^
2	0.453	0.440	133.000	1,147.082	1.142	0.141
3	0.421	0.553	108.000	998.650	1.006	0.467
4	0.378	0.672	85.000	845.565	0.852	0.824
5	0.284	0.784	64.000	687.370	0.687	0.970
6	0.238	0.853	45.000	523.632	0.638	0.968
7	0.234	0.904	28.000	354.000	0.651	0.915
8	0.208	0.957	13.000	178.000	0.620	0.836

** *p <0.01*.

**Figure 2:**
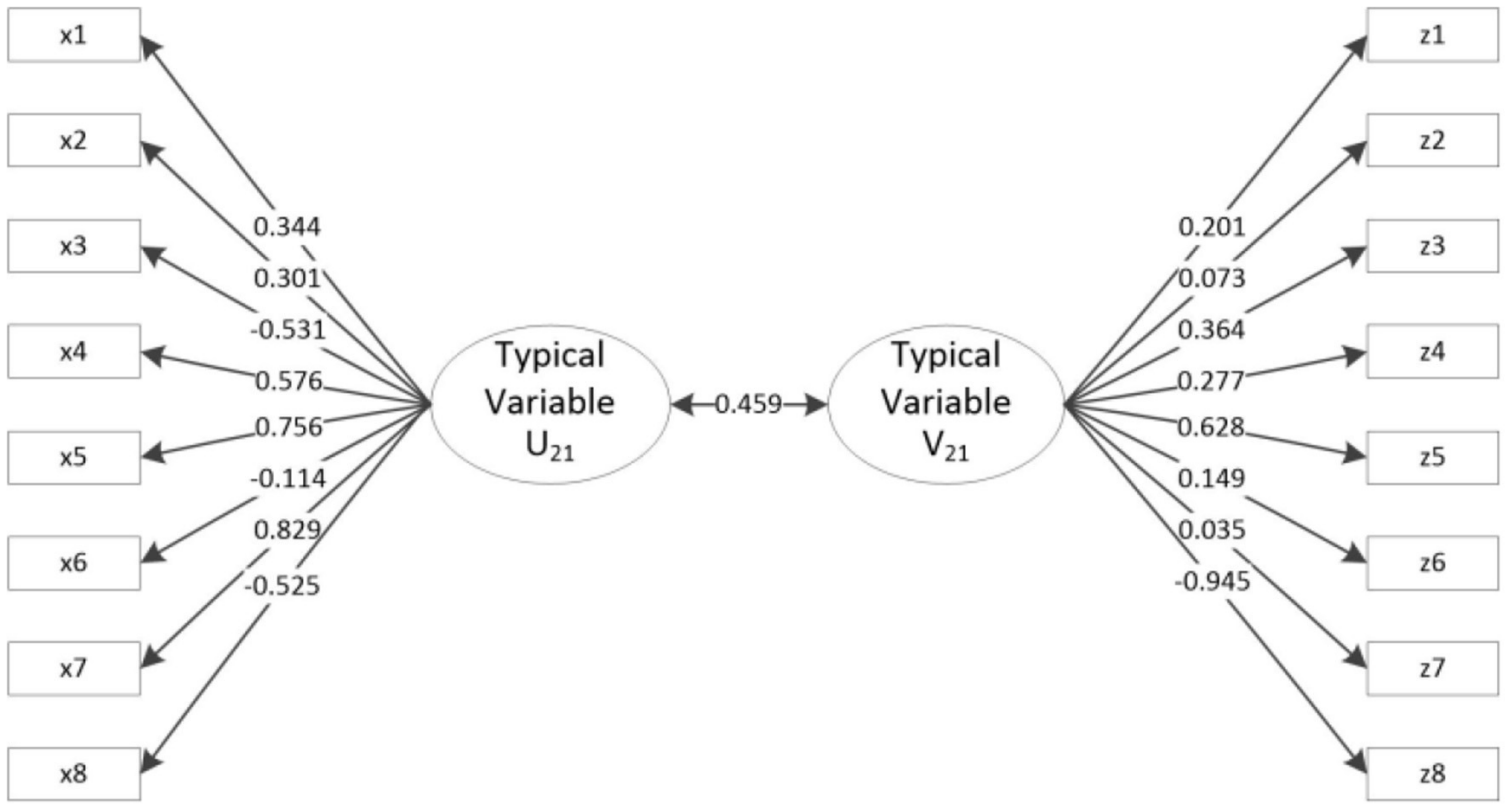
Typical load coefficient and cross-load coefficient of motor performance and motor development.

Table 5 and Figure 2 show the results of the canonical correlation analysis of motor performance and motor development tests. The results show that a total of 8 typical variables are extracted. The *F*-test shows that the first pair of typical variables is significant at the 0.05 level with a correlation coefficient of 0.459 > 0.3, which is a high value. There is a close positive correlation between the first pair of typical variables (U_21_ represents the first typical variable of motor performance, and V_21_ represents the first typical variable of motor development). Focusing on the intragroup difference analysis of typical variables U_21_ and V_21_, the x5 and x7 load coefficients of typical variable U_21_ and Group X are 0.756 and 0.829, indicating a strong correlation, that is, typical variables extract more information from x5 and x7. The load coefficients of z35 and z38 of typical variable V_21_ and Group Z are 0.628 and 0.945 (absolute value), indicating a strong correlation, that is, typical variables extract more information from z35 and z38. Therefore, the main factors of motor performance, i.e., rope skipping (x5) and throw solid ball in place (x7), are closely related to specific skills (z35) and reactions (z38). Namely, there is a very close positive correlation between motor performance (Group X) and motor development (Group Z).

**Table 5:** Typical correlation coefficients and significance of motor behavior and motor development.

**Typical variable pair**	**Canonical correlation coefficient**	**Wilks' lambda**	***df*1**	***df*2**	***F***	***p***
1	0.459	0.638	64.000	1,062.011	1.344	0.040^*^
2	0.304	0.809	49.000	938.559	0.817	0.811
3	0.217	0.891	36.000	815.153	0.601	0.970
4	0.181	0.935	25.000	692.461	0.503	0.980
5	0.152	0.967	16.000	571.932	0.393	0.984
6	0.076	0.990	9.000	457.693	0.210	0.993
7	0.051	0.996	4.000	378.000	0.200	0.938
8	0.041	0.998	1.000	190.000	0.315	0.575

* *p <0.05*.

**Figure 3:**
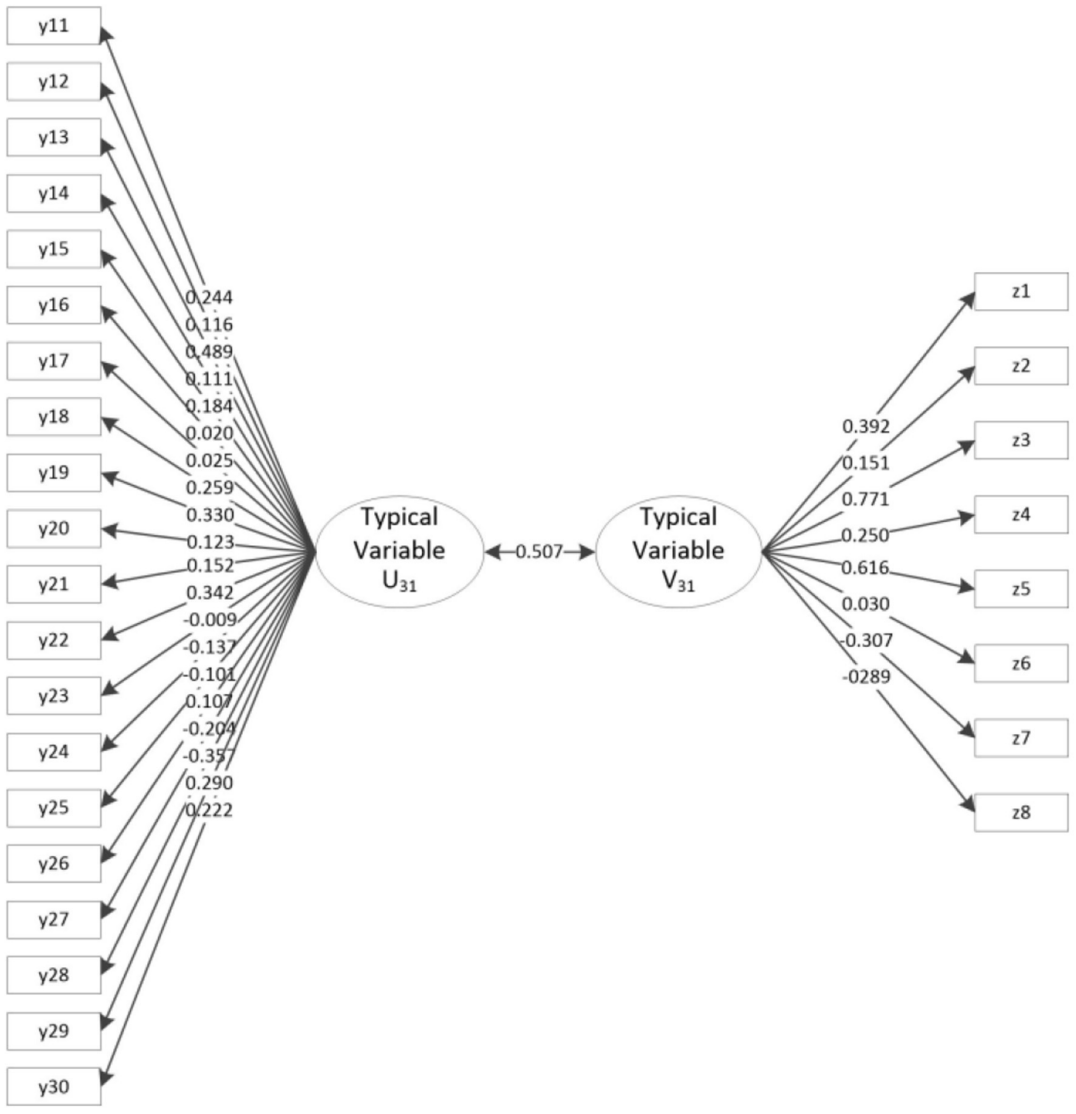
Typical load coefficient and cross-load coefficient of motor behavior and motor development.

Table 6 and Figure 3 show the results of the canonical correlation analysis of motor behavior and motor development tests. The results indicate that a total of 8 typical variables are extracted. The *F*-test finds that the first pair of typical variables is significant at the 0.05 level with a correlation coefficient of 0.507 > 0.5, which is high. There is a close positive correlation between the first pair of typical variables (U_31_ represents the first typical variable of motor behavior, and V_31_ represents the first typical variable of motor development). Focusing on the intragroup difference analysis of typical variables U_31_ and V_31_, the y13 load coefficient of typical variable U_31_ and Group Y is 0.489, indicating a strong relationship, that is, typical variables extract more information from y13. The load coefficients of z33 and z35 of typical variable V_31_ and Group Z are 0.771 and 0.616, indicating a strong relationship, that is, typical variables extract more information from z33 and z35. Therefore, the number per week of interactive activities of more than 20 min (y13) in motor behavior is closely related to the main factors of motor development, i.e., hand-eye coordination (z33) and specific skills (z35). Namely, there is a very close positive correlation between motor performance (Group Y) and motor development (Group Z).

**Table 6:** Typical correlation coefficients and significance of motor behavior and motor development.

**Typical variable pair**	**Canonical correlation coefficient**	**Wilks' lambda**	***df*1**	***df*2**	***F***	***p***
1	0.507	0.395	160.000	1,298.768	1.076	0.047*
2	0.428	0.532	133.000	1,153.690	0.870	0.846
3	0.352	0.651	108.000	1,004.381	0.723	0.984
4	0.305	0.743	85.000	850.399	0.633	0.996
5	0.273	0.820	64.000	691.285	0.563	0.998
6	0.242	0.886	45.000	526.603	0.488	0.998
7	0.176	0.941	28.000	356.000	0.393	0.998
8	0.170	0.971	13.000	179.000	0.411	0.965

*p <0.05.

### Partial Least Squares Regression Analysis of the Tests for Participants With Different Age and Gender

**Figure 4:**
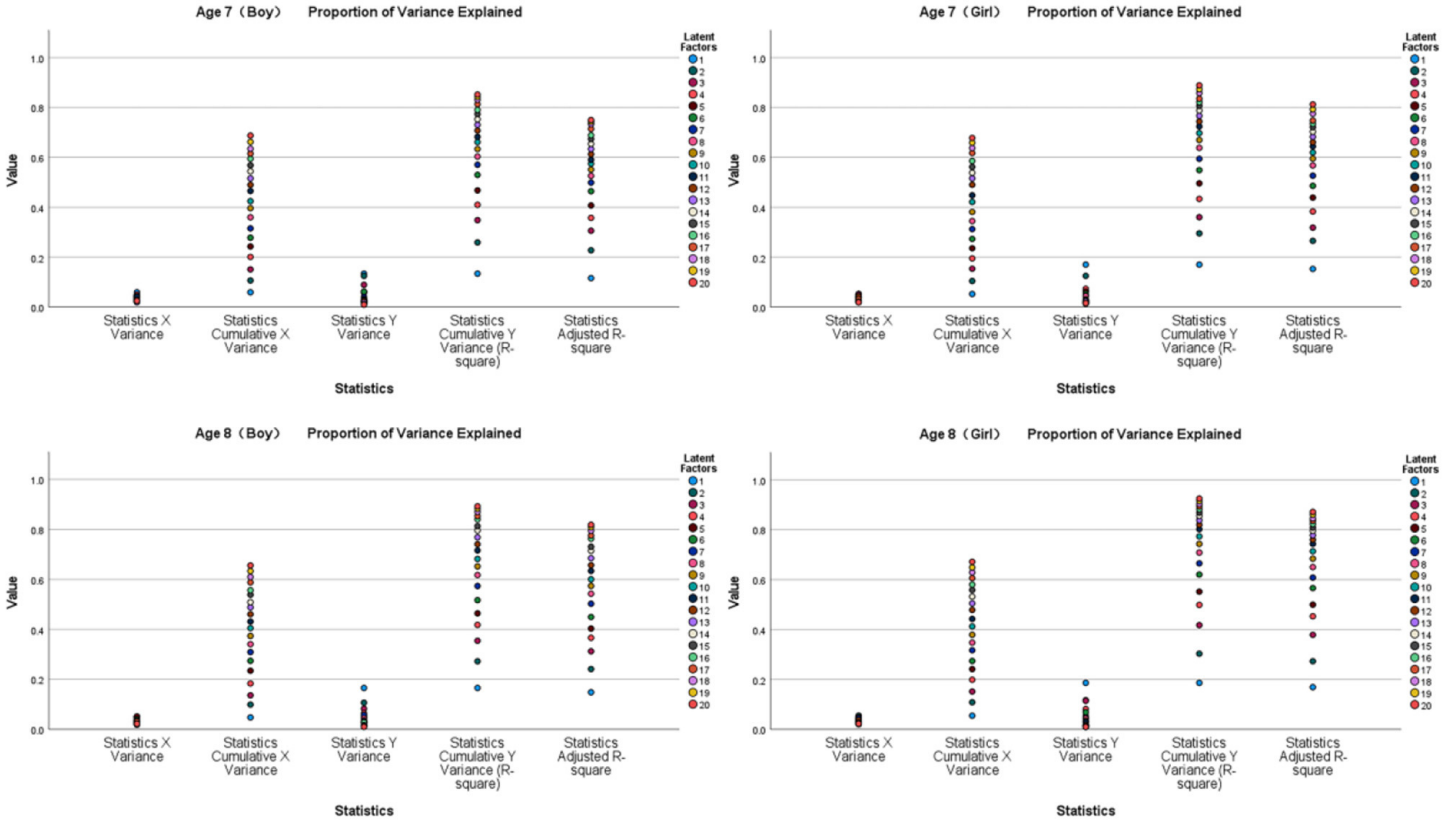
Explanation and analysis of motor behavior on motor performance.

**Figure 5:**
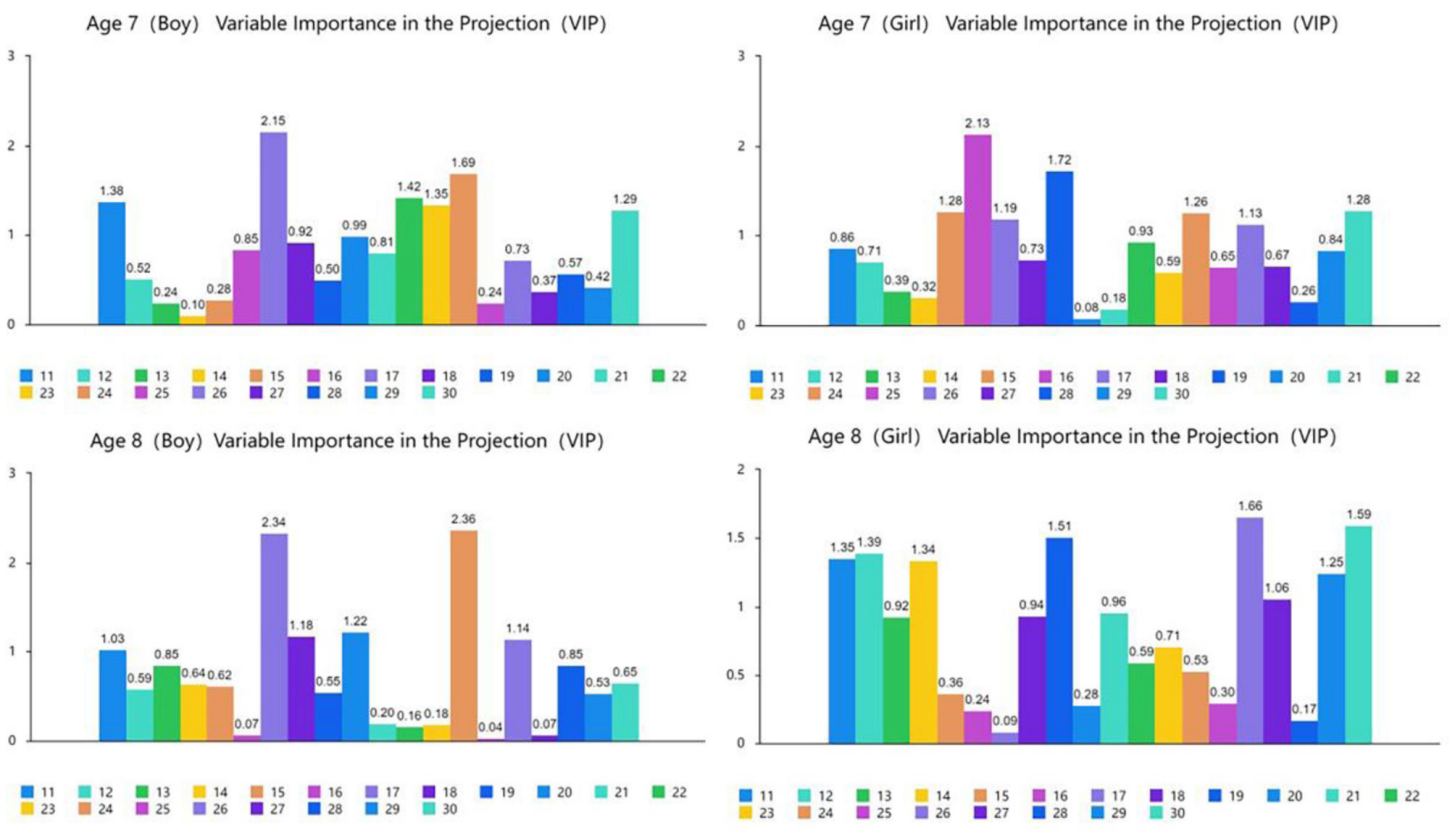
VIP chart of the projection importance index of motor behavior to motor performance.

Figures 4, 5 show the results of the partial least squares regression analysis of the motor behavior and motor performance tests. The results show that the environmental interaction experience of a 7-year-old boy, 7-year-old girl, 8-year-old boy, and 8-year-old girl participating in sports, namely, motor behavior, has a relatively high ability to cumulatively explain motor performance. The explained cumulative variance (R-square) values are 0.852, 0.889, 0.893, and 0.925, respectively, and the adjusted R-square values are 0.750, 0.812, 0.819, and 0.872, respectively. According to the cross-validity analysis, when the Qh^2^ value is >0.0975, the best principal component is one. In this analysis, the most significant indicator of 7-year-old boys' motor performance is y17, followed by y24, y22, y11, y23, and y30. The most significant indicator of 7-year-old girls' motor performance is y16, followed by y19, y15, y30, y24, y17, and y26. The most significant indicator of 8-year-old boys' motor performance is y24, followed by y17, y20, y18, y26, and y11. The most significant index of 8-year-old girls' reaction to motor performance is y26, followed by y19, y12, y11, y14, y29, and y27. Therefore, the main common indicators of 7- to 8-year-old children's motor behavior reflecting motor performance are y11, y17, y19, y24, y26, and y30.

**Figure 6:**
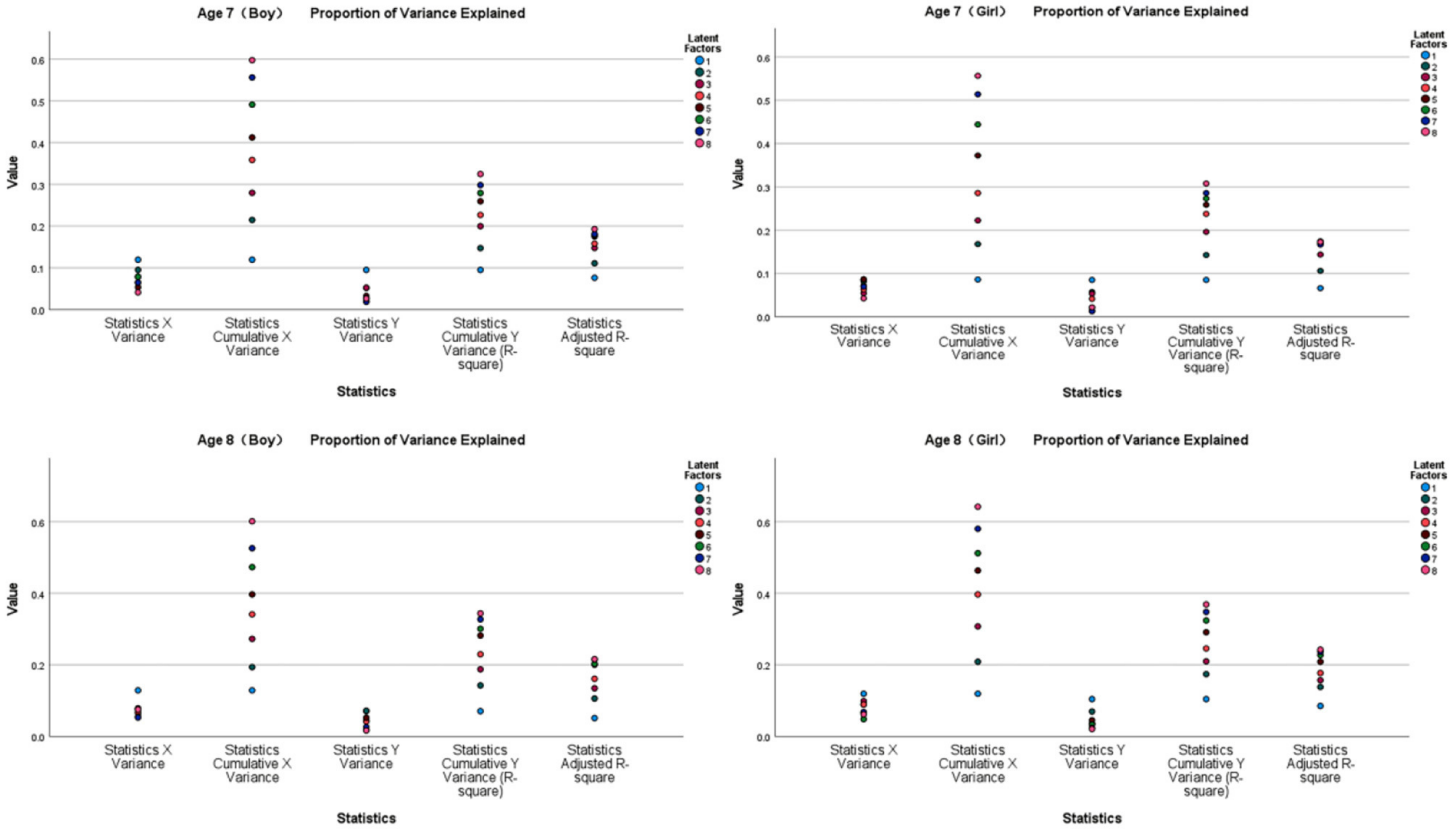
Explanation and analysis of motor development on motor performance.

**Figure 7:**
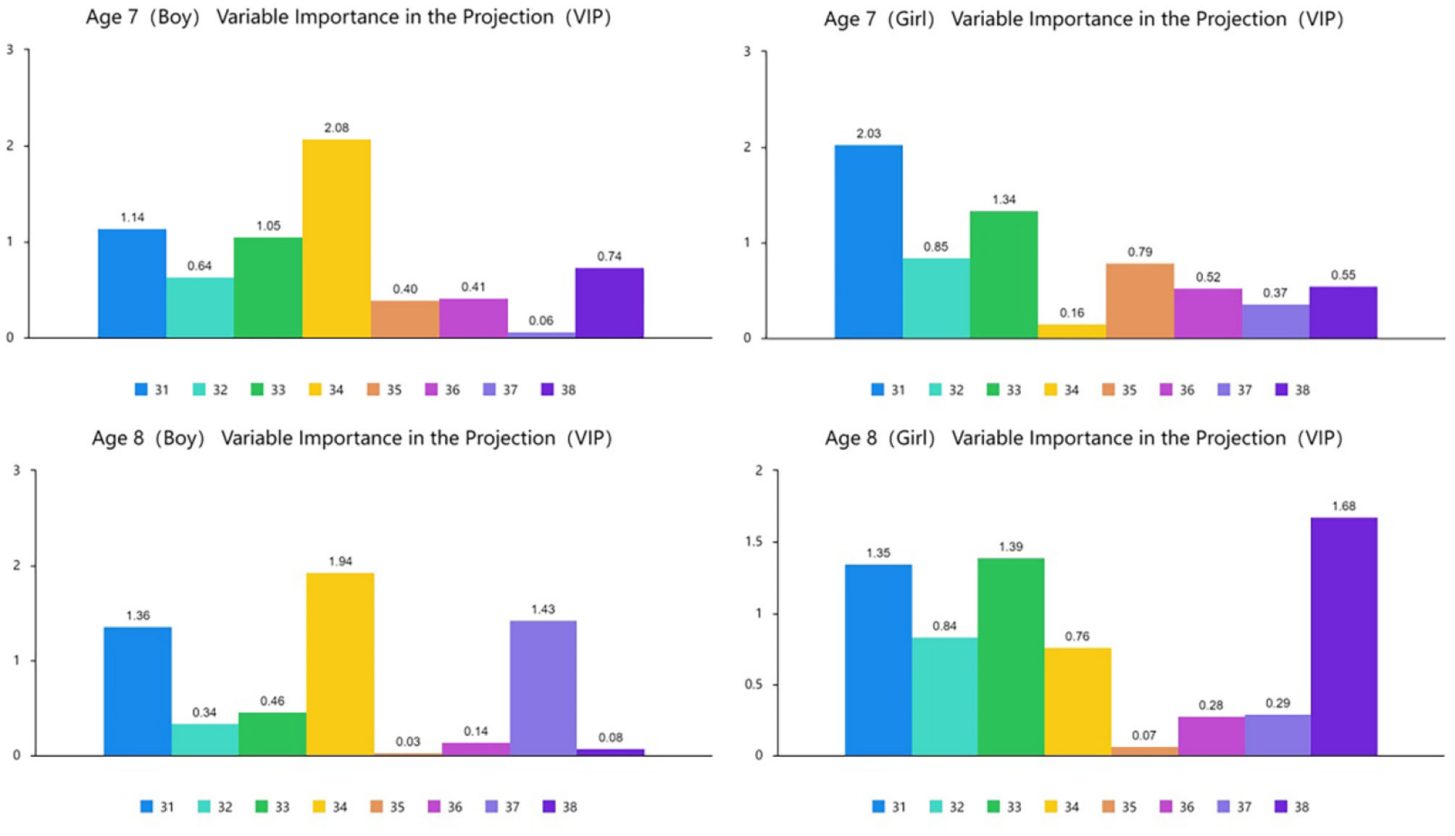
VIP chart of the projection importance index of motor development to motor performance.

Figures 6, 7 show the results of the partial least squares regression analysis of motor development and motor performance tests. The results show that the ability of a 7-year-old boy, 7-year-old girl, 8-year-old boy, and 8-year-old girl to complete motor tasks is related to motor development. The cumulative explanation of motor performance is moderate. The explained cumulative variance (R-square) values are 0.325, 0.308, 0.383, and 0.369, respectively, and the adjusted R-square values are 0.193, 0.173, 0.263, and 0.243, respectively. According to the cross-validity analysis, when the Qh^2^ value is >0.0975, the best principal component is 1. In this analysis, the most significant index reflecting the motor performance of 7-year-old boys is z34, followed by z31 and z33. The most significant index of a 7-year-old girl's reaction to motor performance is z31, followed by z33. The most significant index of an 8-year-old boy's reaction to motor performance is z34, followed by z31 and z37. The most significant index of an 8-year-old girl's reaction to motor performance is z38, followed by z33 and z31. Therefore, z31, z33, and z34 are the main common indicators reflecting the motor performance of children aged 7–8 years.

**Figure 8:**
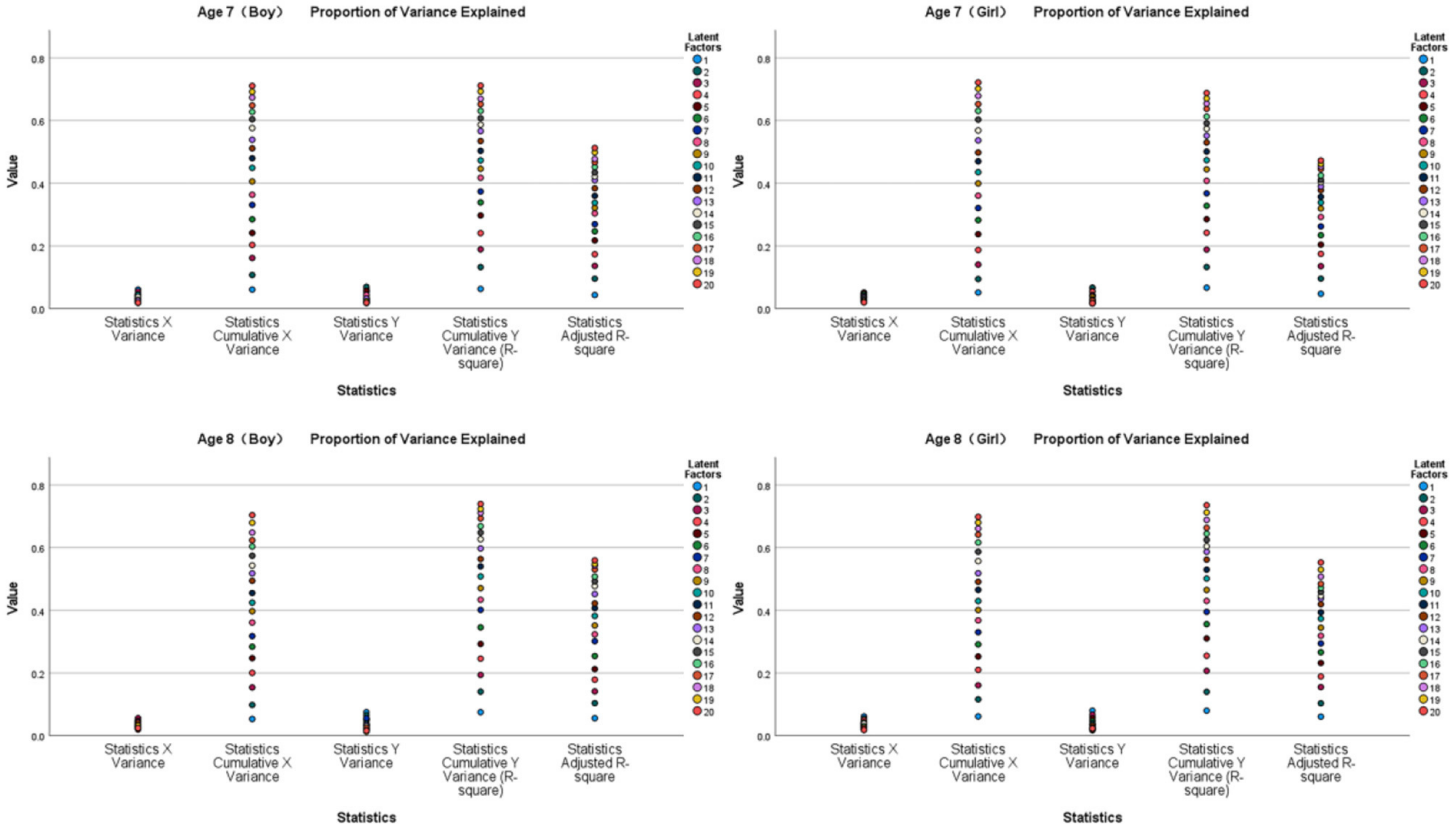
Explanation and analysis of motor behavior on motor development.

**Figure 9:**
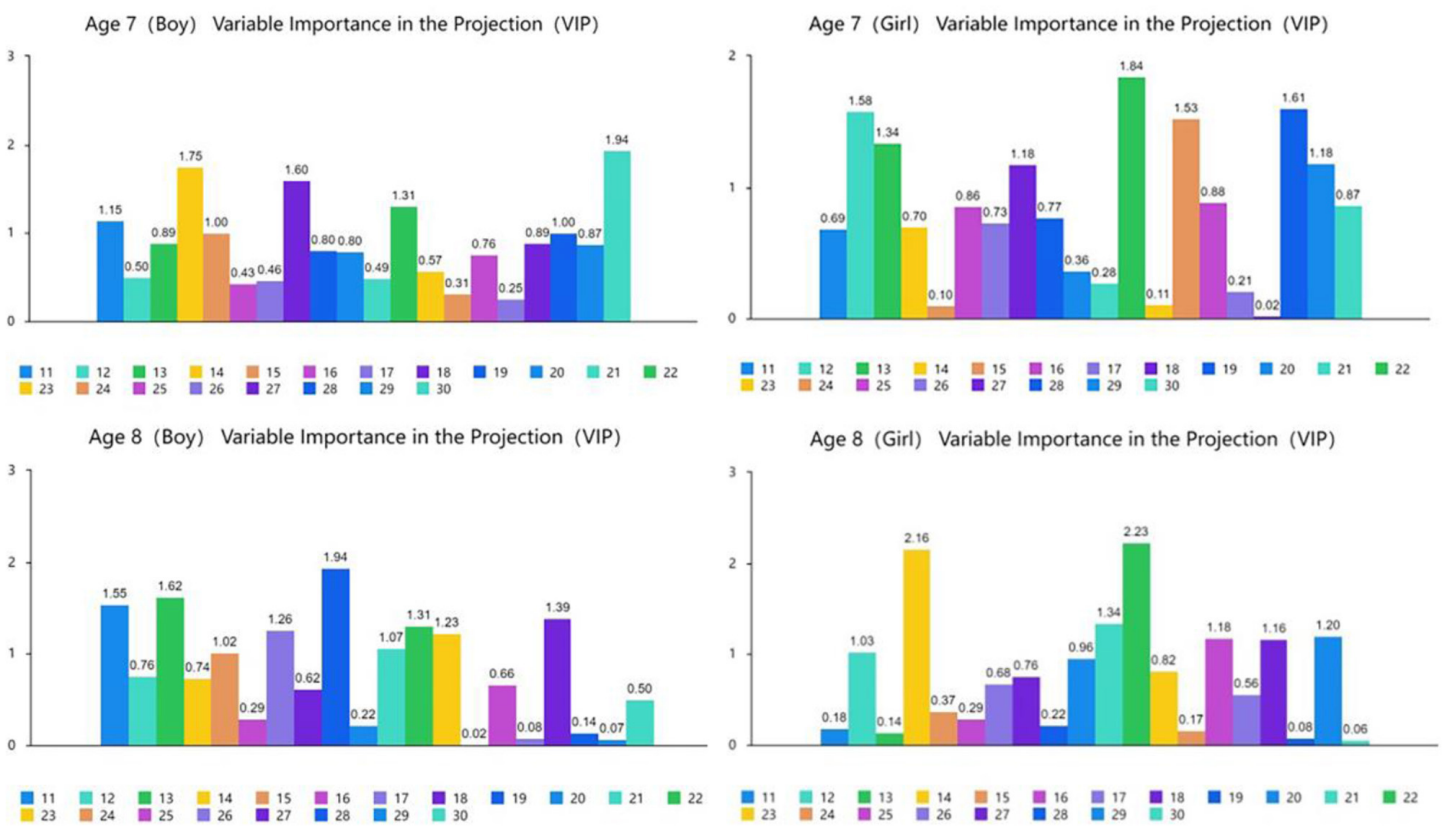
VIP chart of the projection importance index of motor behavior on motor development.

Figures 8, 9 show the results of the partial least squares regression analysis of motor behavior and motor development. The results show that the environmental interaction experience of a 7-year-old boy, 7-year-old girl, 8-year-old boy, and 8-year-old girl participating in sports is motor behavior, and the cumulative interpretation of the ability to complete action tasks for motor development is relatively high. The explained cumulative variance R-square values are 0.712, 0.688, 0.720, and 0.735, respectively, and the adjusted R-square values are 0.513, 0.473, 0.526, and 0.553, respectively. According to the cross-validity analysis, when the Qh^2^ value is >0.0975, the best principal component is one. In this analysis, the most significant index reflecting the motor development of 7-year-old boys is y30, followed by y14, y18, y22, y11, y15, and y28. The most significant index reflecting the motor development of 7-year-old girls is y22, followed by y28, y12, y24, y13, y18, and y29. The most significant index reflecting the motor development of 8-year-old boys is y19, followed by y13, y11, y27, y22, y17, y23, y21, and y15. The most significant index of 8-year-old girls' motor development is y22, followed by y14, y21, y29, y25, y27, and y12. Therefore, the main common indicators of 7- to 8-year-old children's motor behavior reflecting motor development are y11, y12, y13, y14, y15, y18, y21, y22, y27, y28, and y29.

## Discussion

The goal of this study is to design the scales for testing motor performance, motor behavior and motor development of children aged 7–8 years, carry out comparison analysis, and explore the comprehensive evaluation of motor performance and motor development. The canonical correlation analysis results show that there are significant-close positive correlations among the motor behavior, motor development, and motor performance. And the partial least squares regression analysis clarifies that the cumulative interpretation of the motor behavior to motor performance is high, the cumulative interpretation of the motor development to motor performance is moderate, while the cumulative interpretation of the motor behavior to motor development is high. Notably, these findings are applicable for the children in any age (7–8 years) and gender (boy and girl).

Based on the aforementioned overarching results, we further discussed several specific results relating to correlation analysis, impact relationship analysis, and the development of the Motional Quotient scale as well as its theoretical model as follow.

### Correlation Analysis

Most of previous studies focused on the pairwise comparison analysis of motor performance, motor behavior and motor development, while few studies concerned the relationships among these three dimensions. Through the canonical correlation analysis in this study, it's noted that X3 (turning back and running), x5 (rope skipping), x7 (throwing a solid ball in place), y13 (the number of interactive activities per week lasting more than 20 min), y16 (People who feel that they are good at sports are popular), z33 (hand-eye coordination), z35 (special skills), and z38 (reaction) in motor development are the main representative factors. This finding aligns with the physical and mental growth and development standards for Chinese children aged 7–8 years. For example, rope skipping is beneficial to children's fitness, body composition, body health, and body immunity (35–40). Additionally, the rope skipping also have other advantages, such as the improvements in dynamic balance, explosive power (35), speed, agility (38), timing and rhythm, coordination (40, 41) and the contribution to building good self-confidence in sports activities (40). Additionally, after excluding the influence of the data trend, these representative indicators promote each other and are positively correlated; thus, they can be explored as a simplified measurement tool. In particular, x5 (rope skipping), y13 (the number of interactive activities per week lasting more than 20 min) and z35 (specific skills) appeared twice in the correlation analysis, which are more representative. We should pay more attention to these representative indicators for facilitating the physical health conditions of the children aged 7–8 years. Moreover, as previous studies stated, testing the rope skipping helps to identify motor problems in young children, because this skill relies on overall body coordination, and motor Performance (42, 43).

According to Trecroci et al., there are positive changes in balance and motor coordination among preadolescent soccer athletes (41). More and more studies began to link rope skipping with physical education and special training. In practical applications, these indicators focus on improving sports ability through training correct rope skipping and appropriate special technical learning and training to improve the number of interactive activities per week lasting more than 20 min, which is conducive to sports and promotes healthy development. At the same time, education and training related to a child's physical activity (such as speed, strength and sensitivity), motor development and physical skills, as well as the cultivation of self-confidence in activity participation, should be strengthened. The three tests are closely related and can be applied to promote children's sports performance through improved training activities for some indicators. This is consistent with the evidence provided by PDMS for the effectiveness of developmental activities at promoting motor development (30). Moreover, these findings show that continuing to deepen the evaluation of motor performance and development in older children can help provide appropriate education, training and services.

### Impact Relationship Analysis

At the time of childhood, as boys and girls grow, the consequent longer levers and increased muscle tissue have the benefits to increase their strength. Both the boys and girls have the same ability to perform motor skills prior to puberty (44).This study found that the motor behavior of children of different age and gender generally has a high effect of motor performance and motor development, while the effect of motor development on motor performance is moderate. Most of the indicators selected in this study were derived from popular authoritative scales in China and other countries, but it is obvious that some indicators do not play a particular role, rather the cumulative effect of the motor development test on motor performance is moderate. It is assumed that the influence of motor development on motor performance is moderate, but the results of the canonical correlation analysis show that they have a very close positive correlation, and the influence of motor development on motor performance cannot be denied. Therefore, the hypothesis is not reliable. The reason for this result may be that this aspect has not attracted much attention from Chinese children.

It's critical to develop the motor performance for the children (45). For example, successful participation in the structured and non-structured activities, games, and sports demands a certain degree of competence in many fundamental motor skills (46). The main indicators of physical and mental growth reflecting exercise ability are y11 (average daily steps within 28 days), y17 (fear of injury in sports games), y19 (ability to readily learn actions), y24 (exercise only under the influence of partners and not taking the initiative), y26 (examples of health common sense), y30 (understanding of body posture), z31 (posture), z33 (hand-eye coordination), and z34 (behavior). A positive relationship exists between physical and mental growth and motor performance across childhood. These indicators can be used to predict children's motor performance and to focus the formulation of exercise prescriptions for improving motor performance.

The main common indicators of significant motor development reflected by motor behavior are y11 (average daily steps over 28 days), y12 (the number of medium and high intensity exercise sessions per week lasting more than 1 h), y13 (the number of interactive activities per week lasting more than 20 min), y14 (the number of action/skill learning sessions per week lasting more than 10 min), y15 (the amount of action/skill learning for more than 10 min per week), y18 (sports games are more interesting than computers [mobile phones, TV)], y21 (effective strategies are used in sports), y22 (moderate activities enhance will and regulate sleep), y27 (understanding of physical activities), y28 (understanding of movements/skills), and y29 (understanding of activity time and environment). Previous studies clarified that the essence of motor development is behavior development (28), and they also stated the importance of the bodily context in motor development (33). These aforementioned indicators can be used to predict children's motor development. Of these indicators, only y11 is the main indicator of sports behavior reflecting sports ability, which suggests that motor behavior reflects sports development and sports ability with good independence. It is necessary to focus on improving physical activities and behaviors in the environment and tasks to promote behavior and living conditions that are beneficial to sports and health and that meet the measurement requirements of modern medicine's bio-psycho-social health promotion model (47).

### Development of the Motional Quotient Scale and Its Theoretical Model

The ACSM exercise testing and exercise prescription guide is committed to the promotion and integration of sports medicine and sports science in scientific research, education and practical application to maintain and improve physical function, physical fitness, health and quality of life (23). It provides good comprehensive exercise and medical guidance. Currently, the fields of public health and psychology in China mainly focus on the diagnosis of disorders in children, while the field of sports science focuses on the evaluation of teenagers. Physical activities, which are part of the traditional evaluation, generally lack a comprehensive evaluation of sports ability and development.

It is assumed that the three tests can be combined into three dimensions in a comprehensive evaluation scale of motor performance and development.

Cronbach′s alpha internal consistency reliability coefficient was used to evaluate the reliability of the hypothesis scale; the hypothesis scales for 7-year-old boys, 7-year-old girls, 8-year-old boys, and 8-year-old girls were tested. The α coefficients were 0.68, 0.60, 0.67, and 0.64, respectively, suggesting that the internal consistency of the scale was acceptable. The evaluation items of the hypothetical scale were derived from mature scales with good reliability and validity in China and elsewhere, and these items are representative, appropriate and reasonable within the defined scope. Actual measurements, questionnaires and observations yield a reflection of the basic motor performance, motor behavior, psychology and skilled motor performance of individuals, and the evaluation data are accurate and effective. Through expert interviews, 12 experts in related fields agreed that the content of the scale is relatively independent and can comprehensively reflect the development of motor performance in the process of motor development. Therefore, the content validity of the scale is good. The three dimensions of the hypothetical scale, motor performance, motor behavior and motor development, are significantly correlated with each other and have a very close positive correlation.

Therefore, the hypothesis scale is composed of topics with similar content and high statistical correlation. The topics have high correlation and high internal unity. The hypothesis is tenable.

According to the three dimensions of the hypothesis scale, the connotation of the representation hypothesis scale is that “the individual ability to carry out physical exercise, the environmental interactive experience of participating in exercise and the ability to complete action tasks”. Blanche proposed the concept of the “Physical Quotient” in 1930 in addition to the concept of “Physical Age” (48). Mecloy proposed the “Motor Skill Quotient” in 1934 (49), and Anderson identified the “Motor Skill Quotient” in 1948 and applied it to grade evaluation in physical education teaching, which verified the research of the Motor Skill Quotient, however, there was no further explanation (50). Gesell proposed the concept of the “Developmental Quotient” in 1940, which mainly diagnoses the adaptive behavior, gross motor, fine motor, language and personal social behavior of children aged 0–6 years (51). Folio and Fewell published the first commercial PDMS in 1983 (30), which was mainly expressed by the Gross Motor Quotient, Fine Motor Quotient and Total Motor Quotient. In the “Theory of Multiple Intelligences” proposed by Gardner in 1983, Bodily-kinesthetic intelligence was defined as “the coordination and balance ability of human body and the strength, speed and flexibility of movement, which is characterized by the use of physical communication and problem-solving, skilled object operation and activities requiring good motor skills” (52). Craig proposed “Motor Intelligence” in 1990. Research has found that the cognitive processes directly affect the operation efficiency of athletes (53). Linda evaluated physical fitness with the Physical Fitness Quotient in ACSM in 2013 (23). The Physical Fitness Quotient is a comprehensive reflection of healthy physical fitness and skill physical fitness. Most people do not lack IQ (intelligence quotient) and EQ (emotional quotient), but do lack a body awareness ‘quotient”'. Drawing on the research results of the quotient, we explored and extended the research results of many scholars and termed the hypothetical scale the Motional Quotient scale, with an innovation of the physical and motor skills that correspond to the “Emotional” aspect of EQ. The Motional Quotient represents the comprehensive motor ability level of individuals in their age group. From the perspective of the health promotion model, motor performance, as one of the dimensions of evaluation, is comprehensively affected by other dimensions. According to “the bio-psycho-social health promotion model” and children's cognitive development standards (54), the theoretical Motional Quotient model of “The Bio-Behavior-Task” was further constructed to comprehensively evaluate motor performance.

## Conclusions

Through interviews, investigations and quantitative tests, this study designed and validated the scales for testing motor performance, motor behavior, and motor development, respectively, aligning with the physical and mental growth and development standards for Chinese children aged 7–8 years. The three tests are significantly correlated with a very close positive correlation, and, for their high correlations, there are no significant differences in children's age and gender. Moreover, the three tests can be combined and compiled into an MQ (motional quotient) scale to build a theoretical MQ model of “The Bio-Behavior-Task”, which can be a comprehensive motor performance evaluation system in consistent with the standards for students' physical and mental development.

The developed MQ scale helps clarify movement development standards, predict the motor performance and level, achieve predictable development sequence learning, and tailor appropriate sports development plans. In addition, it can be used to set goals and directions for individual sports development, produce highly effectiveness materials to promote sports participation, and provide implementable strategies for promoting health through children's sports.

## Data Availability Statement

The raw data supporting the conclusions of this article will be made available by the authors, without undue reservation.

## Ethics Statement

The studies involving human participants were reviewed and approved by Scientific Ethics Committee of Nanjing University of Science and Technology. Written informed consent to participate in this study was provided by the participants' legal guardian/next of kin. Written informed consent was obtained from the minor(s)' legal guardian/next of kin for the publication of any potentially identifiable images or data included in this article.

## Author Contributions

HZ contributed to data collection and manuscript writing. JC contributed to data interpretation and critical revisions. ZW contributed to the study design. HZ and JC contributed to data analysis. All authors contributed to the article and approved the submitted version.

## Funding

This study was supported by the National Social Science Foundation of China (Grant Number 17CTY016).

## Conflict of Interest

The authors declare that the research was conducted in the absence of any commercial or financial relationships that could be construed as a potential conflict of interest.

## Publisher's Note

All claims expressed in this article are solely those of the authors and do not necessarily represent those of their affiliated organizations, or those of the publisher, the editors and the reviewers. Any product that may be evaluated in this article, or claim that may be made by its manufacturer, is not guaranteed or endorsed by the publisher.
