# Supplementary material for: Relationship Among Motor Behavior, Motor Development, and Motor Performance in Children Aged 7–8 Years in China
Source: Front Public Health. 2022 May 31;10:898266. doi: 10.3389/fpubh.2022.898266 (PMC9194818; doi:10.3389/fpubh.2022.898266)

Appendix 1: Motor development scale

Item	Action procedure	Scoring criteria and Note
Posture (z31)	1) Open your legs, inhale and straighten your arms upward;<br>2) When exhaling, bend the knees slightly downward and keep the arms motionless;<br>3) Squat and return to the initial position;<br>4) The action is repeated three times consecutively.<br>**Shown in Appendix 2: Figure A (a)- (b)**	**Five points:** The movement is performed easily. There is no change in the inclination of the arms, collapse or bowing of the waist, or valgus or varus movement of the knees.<br>**Four points:** The movement is performed relatively easily. The arms show a slight change in inclination, the waist is slightly bent or collapsed, and slight valgus or varus movement of the knees occurs.<br>**Three points:** The movement is performed easily but with arm tilting, waist bowing or collapse, and valgus or varus knees.<br>**Two points:** Inclination of the arms, bowing or collapse of the waist, and valgus or varus knees are obvious.<br>**One point:** Arm tilt, waist bowing or collapse, and valgus or varus knees are very obvious.<br>**Note:** The tester should observe the action from the side and front of the testee.
Operation (z32)	1) Shoot the ball with one hand 2 consecutive times while standing in place, repeat the action 2 consecutive times with the other hand, and repeat again.<br>2) Catch the ball. The tester stands 5 meters from the testee, and throws the ball on a forward and upward trajectory aiming for the testee's head, and the testee quickly catches the ball (**Appendix 2: Figure B(a)**).<br>3) Throw the ball to the ground. After the ball bounces, kick the ball forward more than 5 meters a cross the 3-meter-long line (**Appendix 2: Figure B(b)**).<br>4) Standing 5 meters away from the wall, throw the ball such that it bounces once on the ground before hitting 1-meter-diameter circle on the wall located 1 meter above the ground (**Appendix 2: Figure B(c)**).<br>5) Dribble with control at least 5 times while running at a constant speed across a distance of 5 meters.	One point is awarded for each successfully completed action (step 1-5).
Hand-eye coordination (z33)	1) Swing the ball: hold one ball in one hand, swing the other ball evenly from the central position forward and backward (**Appendix 2: Figure C(a)**) and left and right (**Appendix 2: Figure C(b)**), and then change to the other hand and repeat the action.	One point for each successfully completed action (step1-5).

	2) Rotate the ball: hold the ball in one hand, rotate the other ball at least once, change to the other hand and perform the same action (**Appendix 2: Figure C(c)**).<br>3) Bounce the ball: hold the ball with both hands, place it right in front of the chest, drop the ball with control such that it bounces back higher than the original location, and then change to the other hand and repeat the action (**Appendix 2: Figure C(d)**).<br>4) Catch the ball: hold the ball, bounce the ball at the end of the elastic rope, and catch the bounced ball at a higher position than the held ball (**Appendix 2: Figure C(e)-(f)**).<br>5) Throw the ball: while swinging the ball according to "rotate the ball", let go of the ball (**Appendix 2: Figure C(g)**), and then catch both the rope and ball (**Appendix 2: Figure C(h)**).	**Note:** Ensure no obvious movement of the ball holder, no obvious confusion of the body, uniform swing/ rotation or rebounding of the ball, and gentle movement in the correct direction.
Behavior (z34)	1) Choose any single action or combination of actions that obviously represents a certain labor, activity or behavior skill. The number of actions is participant's age minus 5.<br>2) After reporting the skill name to the tester, the testee performs the imitation.	$\geqslant 2$ excellent (5 points), ≥ 1 excellent (4 points), ≥ 2 good (3 points), ≥ 1 good (2 points), < 1 good (1 point).<br>**Note:** The tester will evaluate the testees' imitation of norms and innovation at three levels, including excellent, good and poor.
Specific skills (z35)	1) Choose one or two optional sports.<br>2) After the testee reports the sports items and activities to the testers, the selected activities are performed.<br>3) Two actions must be completed.<br>4) The testee shows the optional actions to the tester in turn.	$\geqslant 2$ excellent (5 points), ≥ 1 excellent (4 points), ≥ 2 good (3 points), ≥ 1 good (2 points), < 1 good (1 point).<br>**Note:** The tester evaluates the testees' actions at three levels, including excellent, good and poor.
Squat control (z36)	1) Place feet on ground at hip width, wrap the right hand behind the shoulder to touch the back, wrap the left hand from the armpit to the back, and keep the two hands close to the back as much as possible (**Appendix 2: Figures D(a)-(b)**).<br>2) Take a step back with your right foot, lunge with your left foot, and keep your right knee close to but not touching the ground for 5 seconds.<br>3) Retract the right foot and repeat the lunge on the other side, holding the lunge for 5 seconds.	The tester evaluates the testee's movement performance using five levels: very relaxed (5 points), relatively relaxed (4 points), relaxed (3 points), relatively difficult (2 points) and very difficult (1 point).
Dynamic scenario (z37)	1) Choose any 30-second dynamic scenario.<br>2) After reporting the dynamic scenario to the tester, the testee begins to perform. The number of obviously representative actions is the testee's actual age minus 3.	The tester evaluates the testee's expression norms at five levels: excellent (5 points), good (4 points), medium (3 points), qualified (2 points) and unqualified (1 point).
Reaction (z38)	1) The testee walk along a balance beam with a height of 30 cm, a width of 20 cm and a length of 4 m (**Appendix 2: Figures E(a)**).<br>2) Step on the points of a five-pointed star in turn (the distance between two points is 4 m; do not step on two adjacent points, and do not step back) (**Appendix 2: Figures E(b)**).	**Note:** The test time was recorded.

	3) Omnidirectional crawling (front 4 m, right 4 m, back 4 m and left 4 m; any part of the body can move in the next direction when touching the point) (**Appendix 2: Figures E(c)**).<br>4) Rotate 5 turns in place (**Appendix 2: Figures E(d)**).	

Appendix 2: Schematic figures for testing the motor development

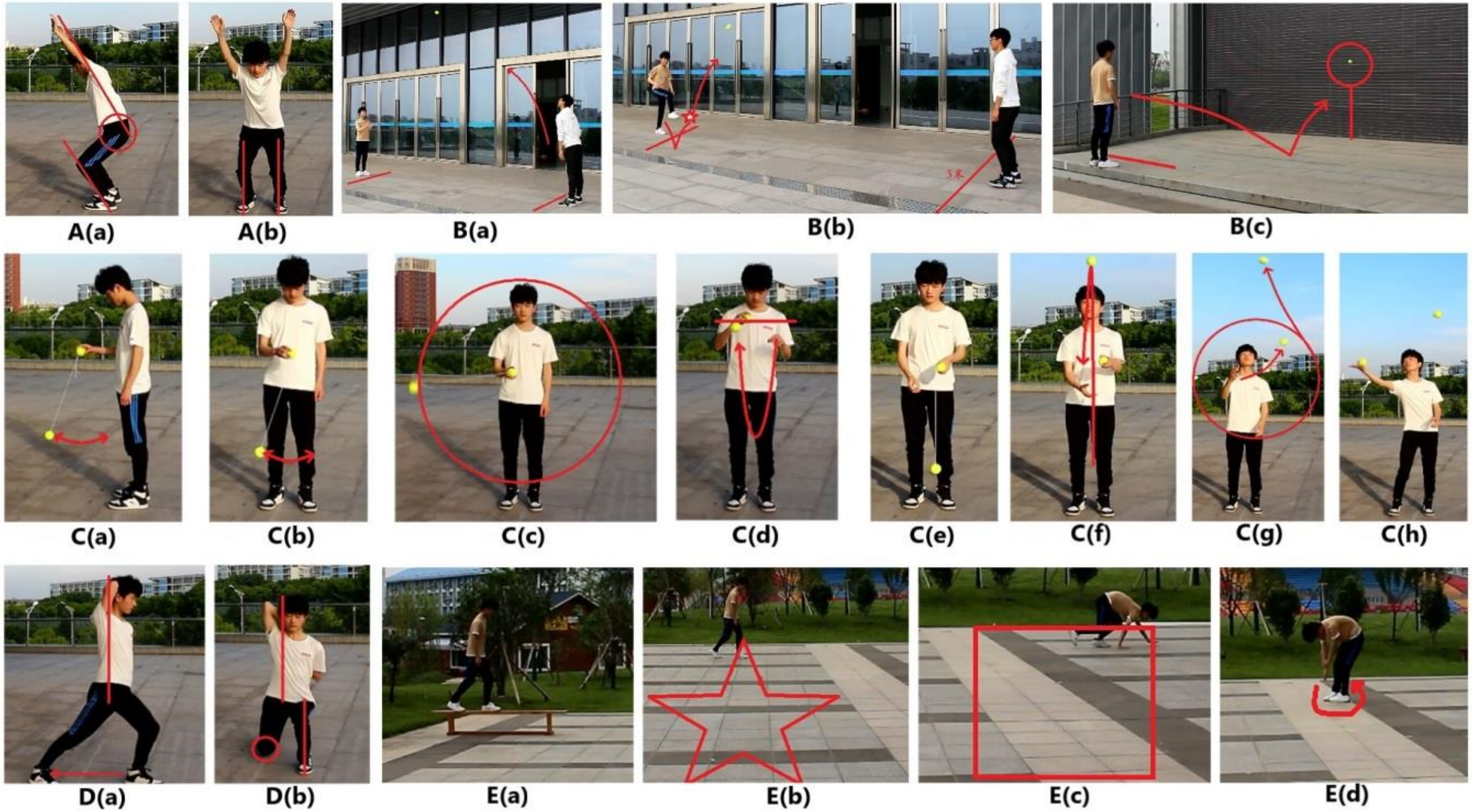

Supplement: Supplementary file 1 [file Data_Sheet_1.pdf]
